# Isolated chronic mucocutaneous candidiasis due to a novel duplication variant of IL17RC

**DOI:** 10.21203/rs.3.rs-3062583/v1

**Published:** 2023-08-01

**Authors:** Kosuke Noma, Miyuki Tsumura, Tina Nguyen, Takaki Asano, Fumiaki Sakura, Moe Tamaura, Yusuke Imanaka, Yoko Mizoguchi, Shuhei Karakawa, Seiichi Hayakawa, Takayo Shoji, Junichi Hosokawa, Kazushi Izawa, Yun Ling, Jean-Laurent Casanova, Anne Puel, Stuart G Tangye, Cindy S Ma, Osamu Ohara, Satoshi Okada

**Affiliations:** Hiroshima University Graduate School of Biomedical and Health Sciences

**Keywords:** Chronic mucocutaneous candidiasis, isolated CMC, IL-17 immunity, IL-17RC, knockout cell line, inborn error of immunity

## Abstract

**Purpose:**

Inborn errors of the IL-17A/F-responsive pathway lead to chronic mucocutaneous candidiasis (CMC) as a predominant clinical phenotype, without other significant clinical manifestations apart from mucocutaneous staphylococcal diseases. Amongst inborn errors affecting IL-17-dependent immunity, autosomal recessive (AR) IL-17RC deficiency is a rare disease with only three kindreds described to date. The lack of an *in vitro* functional evaluation system of *IL17RC* variants renders its diagnosis difficult. We sought to characterize a seven-year-old Japanese girl with CMC carrying a novel homozygous duplication variant of *IL17RC* and establish a simple *in vitro* system to evaluate the impact of this variant.

**Methods:**

Flow cytometry, qPCR, RNA-sequencing, and immunoblotting were conducted, and an *IL17RC*-knockout cell line was established for functional evaluation.

**Results:**

The patient presented with oral and mucocutaneous candidiasis without staphylococcal diseases since the age of three months. Genetic analysis showed that the novel duplication variant (Chr3: 9,971,476–9,971,606 dup (+ 131bp)) involving exon 13 of *IL17RC* results in a premature stop codon (p.D457Afs*16 or p.D457Afs*17). Our functional evaluation system revealed this duplication to be loss-of-function and enabled discrimination between loss-of-function and neutral *IL17RC* variants. The lack of response to IL-17A by the patient’s SV40-immortalized fibroblasts was restored by introducing WT-*IL17RC*, suggesting that the genotype identified is responsible for her clinical phenotype.

**Conclusions:**

The clinical and cellular phenotype of the current case of AR IL-17RC deficiency supports a previous report on this rare disorder. Our newly established evaluation system will be useful for diagnosis of AR IL-17RC deficiency, providing accurate validation of unknown *IL17RC* variants.

## Introduction

Chronic mucocutaneous candidiasis (CMC) is a condition characterized by recurrent or persistent superficial infections caused by *Candida spp*., mainly *Candida albicans* (1–4). Studies of patients with CMC have revealed that interleukin (IL)-17 immunity, which involves T helper 17 (Th17) cells and IL-17 signaling, plays a pivotal role in immune defense against mucocutaneous Candida infections in humans (3, 5). Patients with autosomal dominant (AD) or autosomal recessive (AR) hyper-IgE syndrome due to dominant-negative variants of *STAT3* or *IL6ST* or biallelic variants of *ZNF341* (6–12), AD STAT1 gain-of-function (13–17), AD JNK1 deficiency (18), or AR deficiencies of IL-12p40, IL-12Rβ1, IL-23R (6, 19–21), CARD9 (22–24), or RORγT (25) present with CMC as one of the main infectious manifestations, with complications due to susceptibility to other pathogens, autoimmune manifestations, and/or cancers (4, 26). Such patients are categorized as having syndromic CMC and commonly show reduced circulating Th17 cell proportions due to impaired differentiation, proliferation, and/or survival (4, 5).

Isolated CMC refers to patients with CMC as the predominant clinical phenotype without other significant clinical manifestations (1, 4, 5, 27). However, some patients with isolated CMC may develop mucocutaneous *Staphylococcus aureus* infections (27–29). The discovery of patients with isolated CMC and inborn errors of immunity (IEI) has greatly contributed to our understanding of both the molecular and cellular bases of CMC. Indeed, mono- or biallelic deleterious variants of *IL17F, IL17RA, IL17RC*, and *TRAF3IP2* (encoding *ACT1*) have been detected in patients with isolated CMC, and *MAPK8* has been identified in patients with both CMC and a connective tissue disorder (27–34). These five genes are all directly involved in the IL-17A/IL-17F-dependent IL-17RA/IL-17RC-mediated signaling pathway. Clinical and molecular investigations of these patients suggest that host mucocutaneous immunity against Candida critically depends on IL-17 immunity. IL-17RA is ubiquitously expressed, whereas IL-17RC is predominantly expressed on epithelial and mesenchymal cells (35–37). The two chains form a dimeric receptor for IL-17A and IL-17F homo- or heterodimers, which are mainly produced by Th17 cells (38). IL-17A and IL-17F (IL-17A/F) binding to their receptors results in recruitment of ACT1, an adapter molecule activating several downstream signaling pathways (35, 38), to the cytoplasmic domains of the receptors, leading to release of antimicrobial peptides and cytokines/chemokines such as IL-6, G-CSF, CXCL1 and CXCL8 to control *Candida* infection.

AR IL-17RC deficiency was reported in 2015 in three unrelated patients from three families originating from Argentina and Turkey with isolated CMC and no staphylococcal mucocutaneous manifestations (30). However, additional patients have not yet been reported. Those three patients carried different homozygous nonsense variants, i.e., p.Q138*, p.R376*, and p.Q378*, inherited from heterozygous asymptomatic parents. Fibroblasts from the patients did not exhibit responses to IL-17A/F homo- and heterodimers. We herein report a patient with isolated CMC and a homozygous novel duplication variant of *IL17RC*. In addition, we successfully established a simple *in vitro* system that can accurately assess the functional impact of *IL17RC* variants.

## Methods and Materials

### Molecular Genetics

Genetic testing was performed after obtaining written informed consent from the participants or their guardians. Genomic DNA was extracted from peripheral blood leukocytes and subjected to NGS based gene panel testing for IEI and Sanger sequencing. The detailed method of gene panel testing was described previously (39). Briefly, paired-end sequence libraries were constructed using the TruSeq DNA PCR Free Library Kit (Illumina). The libraries were subjected to 150-bp paired-end sequencing on the HiSeq X Ten system (Illumina). Reads from the fastq files were aligned to the human reference genome GRCh37/hg19 using Burrows-Wheeler Aligner Software. Duplicate reads were removed using Picard Mark Duplicates. The mapped reads around insertions/deletions (Indels) were realigned by the Genome Analysis Toolkit (GATK) Version 4.0. Variant calling was performed using GATK HaplotypeCaller. The called single-nucleotide variants (SNVs) and Indels were annotated using the SnpEff software 4.3.

### Quantitative PCR

Total RNA was extracted from the cell lines with Qiagen RNeasy Mini kit (Qiagen, Hilden, Germany) or SingleShot Cell Lysis Kit (Bio-Rad, Hercules, CA) and reverse transcribed by Superscript III Reverse Transcriptase (Invitrogen, Carlsbad, CA, USA) or iScript Adv cDNA synthesis kit (Bio-Rad, Hercules, CA). All procedures were performed according to the manufacturer’s instructions. Quantitative real-time PCR was performed with Taqman Gene Expression Master Mix (Applied Biosystems) and probes (**Table. S1**) on StepOnePlus Real-Time PCR system (Applied Biosystems). The expression levels of each transcript were determined in triplicate and normalized to the level of *GAPDH*. Data analysis was performed with the comparative threshold cycle method (also known as the 2^−ΔΔCt^ method).

### Immunoblot analysis

V5-taggerd WT and/or *IL17RC* mutant containing plasmids were introduced into SV40-fibroblasts with lipofection, and were collected 24 hours after transfection. For whole-cell extracts, the cells solubilized with sodium dodecyl sulfate (SDS) buffer (100 mM Tris/HCl, pH 6.8, 4% SDS, 20% glycerol and 1.2% 2-mercaptoethanol) and then sonicated. The cell lysates were separated by 10% SDS-PAGE, and proteins were transferred to polyvinylidene fluoride membranes (Merck KGaA, Darmstadt, Germany). After blocking with skimmed milk, the membranes were incubated with primary antibodies against IL-17RC (1:1000, Sigma-Aldrich, HPA019885), V5 (1:1000, Sigma-Aldrich) or β-actin (1:2000, Sigma-Aldrich) at 4°C overnight. Horseradish peroxidase–conjugated goat anti-mouse and anti-rabbit antibodies (GE Healthcare, Buckinghamshire, England) were used as secondary antibodies. Antibody binding was detected with enhanced chemiluminescence reagent (ImmunoStar Zeta reagent, FUJIFILM).

### IL17RC knockout HeLa cell preparation

The CRISPR/Cas9 system was used to generate *IL17RC* knockout HeLa cells. Two sgRNA sequences (GGTGCGTAGGCGCCAGCACG and CTGTGACCTCTGTCTGCGTG) targeting the *IL17RC* gene were designed in exon 3 using CRISPRdirect (40). Cloning of the respective sgRNA tandem constructs into pSpCas9(BB)-2A-GFP (PX458) (Addgene plasmid #48138) was performed according to previous reports and confirmed by sequencing (41). Transfection of sgRNA-Cas9 Plasmid into WT HeLa cells was performed using the Neon^™^ transfection system (Invitrogen/Thermo Fisher Scientific Inc.) according to the manufacturer’s instructions. After 48 hours of incubation from transfection, GFP expressing cells were sorted with FACSAria^™^ III (Becton Dickinson, Franklin Lakes, USA) into single, and expanded under conditions at 37°C, 5% CO2. Successful knockout of *IL17RC* was confirmed by direct sequencing of the genomic DNA and quantitative PCR for *IL17RC* of the candidate clones (Fig.S2).

### CXCL1 quantitative PCR using the IL17RC-targeted HeLa cell line

*IL17RC*-knockout HeLa cells were generated using the CRISPR‒Cas9 system. Briefly, plasmids expressing both pSpCas9 and gRNA designed using exon 13 of *IL17RC* were transfected into WT HeLa cells by electroporation and subsequently sorted into single cells. The *IL17RC*-deficient HeLa cells were plated in 96-well plates at a density of 1.6 × 10^4^ cells/well and incubated for 24 hours and then transfected with mock, pUNO1 vector, WT or *IL17RC* mutant plasmid (20 ng). After 24 hours, the transfected cells were left unstimulated or stimulated with 100 ng/mL recombinant human IL-17A for 4 hours. cDNA was synthesized using total RNA extracted from the cells, and quantitative PCR was performed for *CXCL1*. Expression level of each transcript was determined in triplicate and normalized to the level of *GAPDH*. Data analysis was performed with the comparative threshold cycle method (also known as the 2^−ΔΔCt^ method).

### Isolation and functional characterization of human T cells

Naïve and memory CD4^+^ T cells were isolated by excluding Tregs (CD4^+^CD25^hi^CD127^lo^) then sorting CD45RA^+^CCR7^+^ cells and CD45RA^−^CCR7^+/−^ cells, respectively. Purity of the recovered populations was > 98%. Sorted naïve and memory CD4^+^ T cells were cultured at a density of 150–200×10^3^ cells/ml in 96-well round-bottom plates with T cell activation and expansion (TAE) beads (anti-CD2/CD3/CD28; Miltenyi Biotech) alone (Th0) or under Th1 (50 ng/ml IL-12; R&D systems), Th2 (1 U/ml IL-4; DNAX), or Th17 (10 ng/ml TGF-β1; PeproTech, 200 ng/ml IL-1β; PeproTech, 200 ng/ml IL-6; PeproTech, 200 ng/ml IL-21; PeproTech, and 200 ng/ml IL-23; PeproTech) polarizing conditions. After 5 days, supernatants were harvested to measure secretion of IL-2, IL-4, IL-5, IL-13, IL-17A, IL-17F, IFN-γ and TNF-α using cytometric bead arrays (Becton Dickinson). IL-22 secretion was measured by ELISA (PeproTech). Intracellular cytokine expression was determined following re-stimulation of cells with phorbol 12-myristate 13-acetate (Sigma-Aldrich) and ionomycin (Sigma-Aldrich) with addition of Brefeldin A (10μg/ml) after 2 hours.

Other materials and methods are described in the “Supplementary Materials”.

## Results

### A sporadic case of CMC

We examined a seven-year-old girl born to Japanese parents from the same city but without any known consanguinity (Fig. 1A). The prenatal history was normal, and there was no family history of IEI. From the age of three months onward, the patient developed oral and cutaneous candidiasis, especially at the external eye angle. (Fig. 1B). Mucositis of the nasal cavity and vulva was also observed. These symptoms of oral and cutaneous candidiasis responded well to fluconazole treatment, and flare occurred with discontinuation of treatment. Endoscopy examination at the age of seven years revealed that she had esophageal candidiasis (Fig. 1B). Antifungal drugs had never been used continuously due to the concern of emerging resistant strains. Her oral and cutaneous candidiasis therefore persisted, though she never developed invasive candidiasis. She had sensitive skin and recurrent sweating rashes, which especially grew worse in summer. Mosquito bites were abnormally swollen, sometimes resembling impetigo. Her growth and development were normal, including hair, nails, teeth, and sweating. She also developed herpes zoster of the left shoulder and right side of the head at the age of six years. Otherwise, she had no episodes indicating susceptibility to severe bacterial or viral diseases. Immunological investigations were unremarkable (**Table. S2**). She was thus given a diagnosis of isolated CMC based on the absence of any phenotypes other than CMC.

### Identification of a homozygous novel duplication variant of IL17RC

We performed next-generation sequencing (NGS) gene panel testing for IEI using genomic DNA from the patient’s leukocytes, revealing an increased number of reads in a region including the entire exon 13 of *IL17RC* (Fig. 1C). This finding was not detected by the variant calling system but was identified manually when we directly analyzed the data aligned to the reference genome. Subsequent Sanger sequencing of the patient’s DNA identified a homozygous novel duplication variant (Chr3: 9,971,476–9,971,606 dup(+131bp), c.1324_1372dup) of *IL17RC* (Fig. 1D). A genetic test of the patient’s healthy mother and siblings revealed them to be heterozygous for the variant or wildtype (Fig. 1D). A genetic test was not available for the father. This duplication variant was not found in various public databases, such as gnomAD (https://gnomad.broadinstitute.org, v2.1.1) or TOPMED/BRAVO (https://bravo.sph.umich.edu), suggesting that it is a novel and private variant. Analysis of mRNA extracted from the patient’s fibroblasts revealed that this variant results in a duplication of 46 (exon13dup_1) or 49 (exon13dup_2) bases *IL17RC* of exon 13 (Figs. 1E, S1). Both the 46- and 49-base duplications in exon 13 cause a frameshift resulting in a premature stop codon (p.D457Afs*16 or p.D457Afs*17) (Fig. 1E). This variant affects a conserved SEFIR (similar expression to fibroblast growth factor genes and IL-17R) domain of IL-17RC, which is essential for signaling to ACT1 (42) (Fig. 1F). Additionally, the variant has a high CADD (combined annotation-dependent depletion) score of 33 (Fig. 1G). Thus, the identified variant is predicted to be loss-of-function. Collectively, these data suggest that this novel homozygous duplication variant of *IL17RC* is causal for the patient’s isolated CMC.

### Evolutionary and epidemiological genetics of AR IL-17RC deficiency

**Figure 2:**
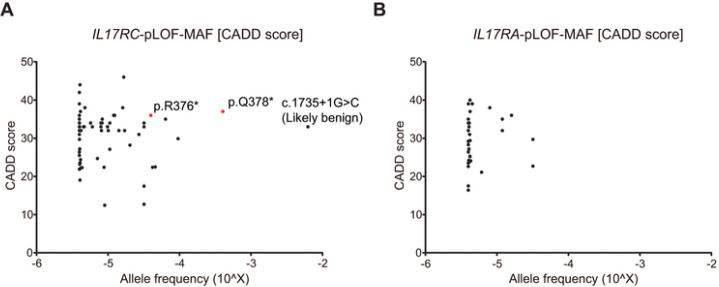
Epidemiological genetics of IL17RC and IL17RA. **A and B,** MAF and CADD scores (GRCh37-v1.6) for pLOF variants of the (A) *IL17RC* and (B) *IL17RA*genes in the gnomAD database (v2.1.1). The red dot shows previously reported *IL17RC*-LOF variants.

Among IEI causing isolated CMC, AR IL-17RA deficiency has been reported thus far in 28 cases from 16 kindreds, but AR IL-17RC deficiency has been reported in only four cases, including the current case (Case summary: **Tables S3, S4**) (27, 29, 30, 43–45). We therefore focused on causes of the difference in the frequency of AR IL-17RC deficiency and AR IL-17RA deficiency. We first investigated genomic selection indices to estimate the strength of negative selection at the *IL17RA* and *IL17RC* loci. The consensus score for negative selection (CoNeS), as developed with the combination of several intraspecific and interspecific statistics, was calculated to be 0.73 (top 76.7%) and 1.09 (top 86.9%) for *IL17RA* and *IL17RC*, respectively (**Table S5**) (46). Furthermore, the gene damage index (GDI) values were 11.0 (top 92.0%) and 6.62 (top 78.2%) for *IL17RA* and *IL17RC*, respectively (47). These results indicate that both *IL17RA* and *IL17RC* are not under strong negative selection, with no obvious difference in their selection pressure. Next, we analyzed the frequency of predicted loss-of-function (pLOF) variants in gnomAD. The cumulative frequency of pLOF variants is lower in *IL17RA* than in *IL17RC*, at 1.70×10^−4^ and 1.22×10^−3^, respectively, even after filtering out a likely benign variant (Fig. 2B). Intriguingly, an individual harboring homozygous p.Q378* in *IL17RC*, a variant previously reported to cause isolated CMC, is listed in gnomAD (n = 1, allele frequency = 4.03×10^−4^), whereas no homozygous individual for pLOF variants in *IL17RA* was found (Fig. 2A).

### Abnormal IL17RC mRNA and protein expression in patient fibroblasts

**Figure 3:**
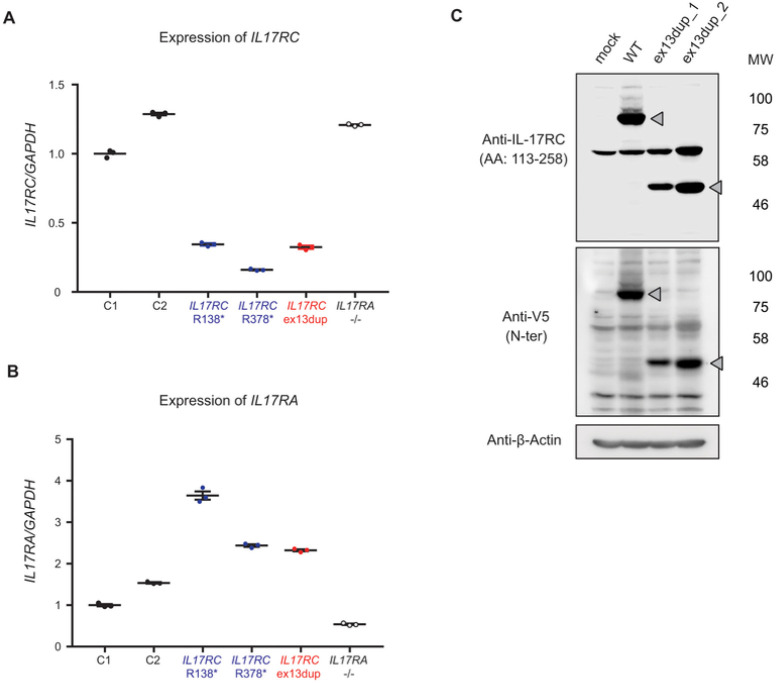
Impaired production of *IL17RC*mRNA and protein with the *IL17RC* duplication mutant allele. **A and B,** mRNA levels of *IL17RC* (A) and *IL17RA*(B) in SV40-immortalizedfibroblasts from controls, the patient, IL-17RC-deficient individuals (R138* and R378*) and an IL-17RA-deficient individual, as detected by the TaqMan assay. *GAPDH* was used for normalization as an endogenous control. Error bars represent SEM (n = 3). The results shown are representative of three independent experiments. Statistical analysis was performed using one-way ANOVA with Tukey’s post hoc test. *: *p* < 0.001. **C,** Immunoblotting of total protein extracts from patient fibroblasts transfected with the empty pUNO1mcs vector (mock) or the pUNO1 plasmid encoding the WT or exon13dup IL-17RC protein. IL-17RC was detected with an anti-IL-17RC antibody (recognizes amino acids 113–258) or an antibody against the N-terminal V5-tag. Gray triangles represent the targeted bands. These experiments were repeated at least three times.

We first assessed levels of *IL17RC* mRNA by RT-qPCR in the patient’s SV40-immortalized fibroblasts. We found decreased *IL17RC* but not *IL17RA* mRNA expression in her fibroblasts compared to cells from healthy controls or from a patient with AR IL-17RA deficiency (27) (Fig. 3A, 3B). These results are comparable to findings for fibroblasts from previously reported patients with homozygous variants (p.Q138*, p.Q378*) of *IL17RC* (30). We next assessed the level of IL-17RC protein expression in the patient’s fibroblasts. As in a previous report, immunoblot analysis failed to identify a band specific for IL-17RC in SV40-immortalized fibroblasts from healthy controls and the above-mentioned patients, possibly due to low expression levels of IL-17RC (30). Therefore, we transiently transfected the fibroblasts with an empty vector or expression plasmid containing wildtype (WT) or exon13dup *IL17RC* and then assessed protein expression. Cells transfected with the vector plasmid WT IL-17RC showed a band at the expected molecular weight (MW) of 86 kDa corresponding to IL-17RC isoform 1 when using a polyclonal antibody directed against amino acids 113–258 of IL-17RC (Fig. 3C). In contrast, a truncated band of approximately 52 kDa was detected in cells transfected with the plasmid encoding exon13dup *IL17RC* (Fig. 3C, top panel). Similar findings were obtained using an antibody against the N-terminal V5-tag (Fig. 3C, bottom panel). These results suggest that the duplication variant of *IL17RC* identified in the present patient impacts both mRNA and protein IL-17RC levels.

### Establishment of a functional validation system using CXCL1 quantitative PCR for IL17RC variants

**Figure 4:**
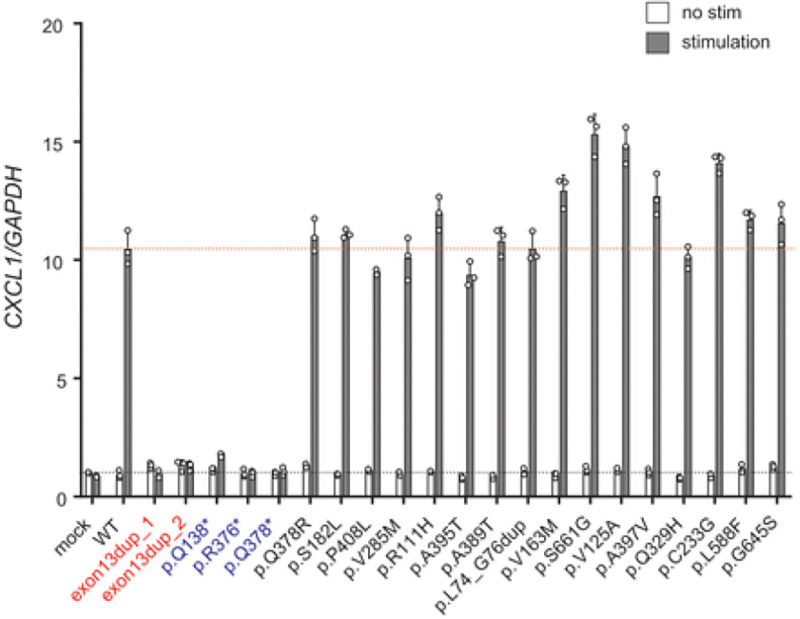
New *in vitro* system for functional validation of *IL17RC* variants. **A,** Induction of *CXCL1* expression in *IL17RC*-knockout HeLa cells transfected with an empty vector (mock) or an IL-17RC plasmid encoding WT or each of the mutants (exon13dup_1, 2, Q138*, R376*, Q378*, or polymorphisms reported in public databases) after 4 hours of stimulation with 100 ng/mL IL-17A, as determined by the TaqMan assay. *GAPDH* was used for normalization as an endogenous control. The results shown are representative of three independent experiments. Error bars represent SEM (n = 3).

Together with the development of NGS approaches, establishing appropriate evaluation systems for variants of unknown significance has become imperative. However, to date, there is no simple *in vitro* system to validate the pathogenicity of *IL17RC* variants. Currently, patient-derived fibroblasts are used to functionally assess the impact of *IL17RC* variants (30). We thus established an *in vitro* system to functionally assess the impact of *IL17RC* variants by measuring induction of *CXCL1* expression by RT-qPCR in an *IL17RC*-knockout HeLa cell line after 4 hours of stimulation with IL-17A (100 ng/mL). We selected HeLa cells because they are immortalized epithelial tissue cell lines expressing IL-17RA and IL-17RC. The *IL17RC* gene was knocked out using the CRISPR/Cas9 system (for details, see Methods and Materials, **Fig. S2**). In this *IL17RC*-knockout HeLa cell system, no *CXCL1* expression was induced after IL-17A stimulation of mock cells, whereas strong induction was observed upon after IL-17A stimulation of cells transfected with WT *IL17RC*. Similarly, cells transfected with any of the gnomAD variants with an MAF > 0.001 or found at a low frequency (MAF ≤ 0.001) but with a CADD score > 24 induced high levels of *CXCL1* expression after IL-17A stimulation (Fig. 1G, 4). In marked contrast, no *CXCL1* expression was induced when cells transfected with any of the previously or currently identified *IL17RC* mutant alleles were stimulated with IL-17A (Fig. 4), further demonstrating the deleteriousness of these alleles. These results show the value of such a system in assessing the impact of novel *IL17RC* variants, clearly discriminating between loss-of-function and neutral *IL17RC* variants. Collectively, these findings demonstrate that the novel duplication variant of *IL17RC* identified in the current study is functionally deleterious.

### Patient fibroblasts showed abolished responses to IL-17A

**Figure 5:**
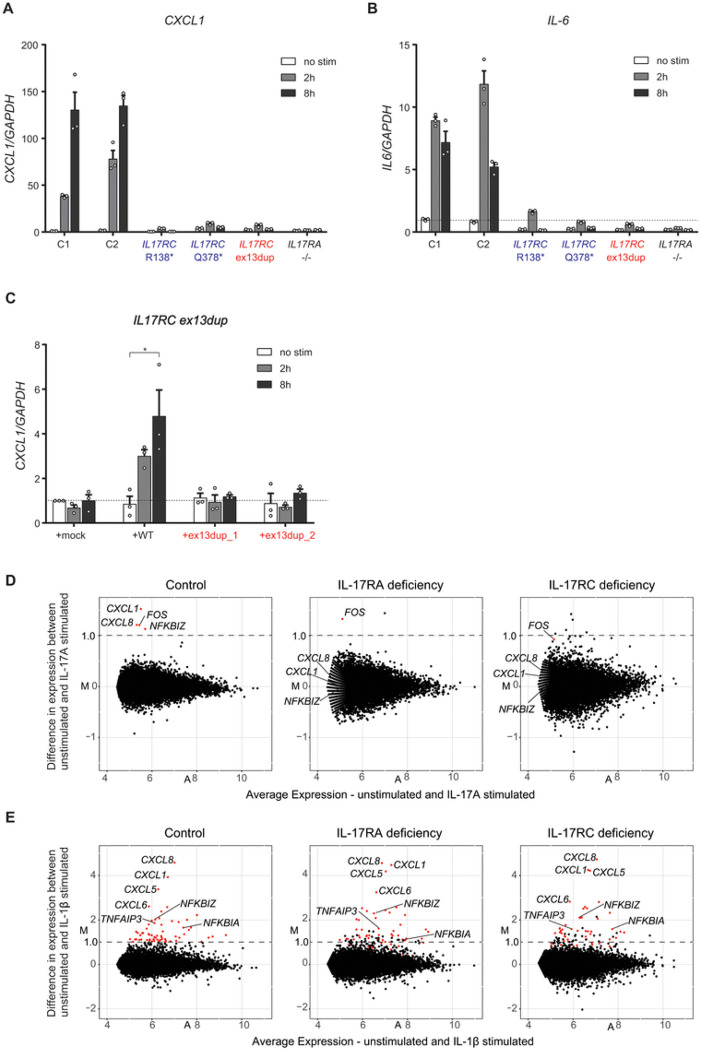
Patient-derived SV-40-immortalized fibroblasts fail to respond to IL-17A stimulation. **A and B,** Induction of *CXCL1* (A) and *IL6* (B) expression in SV40-immortalized fibroblasts from controls, the patient, IL-17RC-deficient individuals (R138* and Q378*) and an IL-17RA-deficient individual after 2 or 8 hours of stimulation with 100 ng/mL IL-17A, as determined by the TaqMan assay. The results are representative of three independent experiments. Error bars represent SEM (n = 3). **C,** Complementation of fibroblasts from patients with the WT *IL17RC* allele. *CXCL1*induction by SV40-immortalized fibroblasts from the patient transfected with an empty vector (mock) or an IL-17RC plasmid encoding WT or exon13dup mutants after 2 or 8 h of stimulation with 100 ng/mL IL-17A, as determined by the TaqMan assay. *GAPDH* was used for normalization as an endogenous control. The data shown represent mean values from three independent experiments. Error bars represent SEM (n = 3). Statistical analysis was performed using one-way ANOVA with Dunnett’s multiple comparison test. *: *p* < 0.05. **D and E,** MA plot analysis of RNA sequencing data of SV40-immortalized fibroblast from controls, the patient, and an IL-17RA-deficient individual. MA plot showing log2-transformed average signal versus log2-fold change by IL-17A (D) or IL-1β (E) stimulation in each cell. Red dots show genes with upregulated expression (>1.0) in fibroblasts from controls (n = 2). The X-axis showsaverage expression (unstimulated and IL-17A or IL-1β stimulated), and the Y-axis represents the difference in expression between unstimulated and IL-17A- or IL-1β-stimulated cells.

We next performed RT-qPCR to assess the response of patient fibroblasts to IL-17A. IL-17A stimulation (100 ng/mL for 2 or 8 hours) induced expression of both *CXCL1* and *IL6* mRNA in fibroblasts from healthy controls but not from the patient carrying the homozygous duplication variant of *IL17RC* (Fig. 5A, B). These results are similar to those for fibroblasts from previously reported patients with homozygous *IL17RC* (p.Q138*, p.Q378*) (30) or *IL17RA* (p.Q284*) (27) variants (Fig. 5A, 5B). Furthermore, transfecting patient fibroblasts with a plasmid encoding WT but not mutant IL-17RC (isoform 1) restored the response to IL-17A (Fig. 5C). Conversely, the response to IL-1β was normal in all of these cells (**Fig. S3A**). Taken together, the lack of response to IL-17A in the patient’s SV40-immortalized fibroblasts was rescued by WT IL-17RC expression, confirming this variant to be the genetic cause of her isolated CMC. We then used 3’-RNA sequencing to further investigate the molecular and functional impact of AR IL-17RC deficiency in response to IL-17A. IL-17A stimulation of healthy control SV40-immortalized fibroblasts upregulated IL-17 signaling pathway-related genes, such as *CXCL1*, *CXCL8*, and *NFKBIZ* (Fig. 5D). In contrast, no changes in expression of these genes were observed in the patient’s fibroblasts or in fibroblasts from an IL-17RA-deficient patient following stimulation with IL-17A (27) (Fig. 5D). IL-1β stimulation upregulated the same pattern of cytokine response-related genes among fibroblasts from healthy subjects and IL-17RC-deficient and IL-17RA-deficient patient (Fig. 5E). Enrichment analysis of these upregulated genes showed that the same Gene Ontology terms were enriched in both the control and patient cells, indicating no molecular differences in response to IL-1β (**Fig. S3B**). These data suggest that AR IL-17RC deficiency, similar to AR IL-17RA deficiency, abolishes IL-17A signaling but that these defects do not affect the IL-1β signaling pathway.

### Normal differentiation of IL-17-producing T cells and secretion of IL-17 cytokines

**Figure 6:**
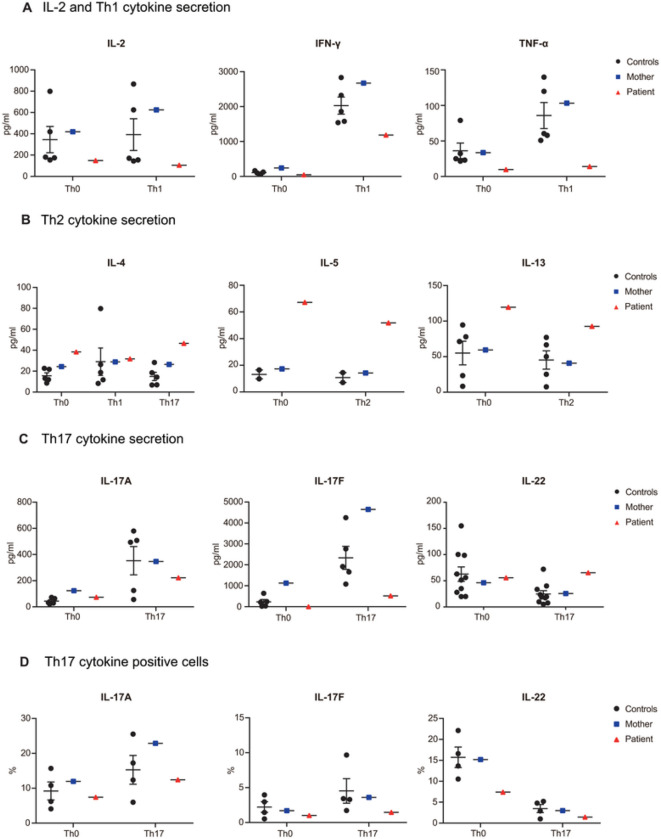
*In vitro* functional characterization of CD4^+^ Th cells with AR IL-17RC deficiency. **A-D,** Secretion of IL-2, Th1 (IFN-γ and TNF) (A), Th2 (IL-4, IL-5, and IL-13) (B), and Th17 (IL-17A, IL-17F, and IL-22) (C) cytokines by memory CD4^+^ T cells and frequency of Th17 (IL-17A, IL-17F, and IL-22) cytokine-positive memory CD4^+^ T cells (D) after 5 days of stimulation with T-cell activation and expansion beads (anti-CD2/CD3/CD28) alone (Th0) or under Th1 (IL-12)-, Th2 (IL-4)-, or Th17 (TGF-β, IL-1β, IL-6, IL-21, and IL-23)-polarizing conditions. Error bars represent SEM. Black dots show healthy controls, blue squares show the heterozygous carrier mother, and red triangles show the patient with AR IL-17RC deficiency.

We next performed deep immunophenotyping and *in vitro* functional experiments to investigate the detailed immune cell biology of our patient. The numbers and frequencies of her total B cells and B-cell subsets were similar to those in healthy donors (**Fig. S4A-E**). Consistent with intact *in vitro* B-cell maturation *in vivo*, the ability of naïve B cells from the patient to undergo proliferation, Ig class switching, and plasma cell generation in response to T-dependent and T-independent stimuli *in vitro* were also similar to those in healthy controls (**Fig. S4F-H**). Furthermore, frequencies of CD4^+^ T-cell subsets, such as Th1, Th17, T follicular helper (Tfh), and regulatory T (Treg) cells, were normal, except for a higher frequency of Th17-phenotype cells among all Tfh cells compared to healthy controls (**Fig. S5A-C, S5E-G**). The frequencies of CD8^+^ T-cell subsets were also similar to those in healthy donors (**Fig. S5B, S5D**). Under *in vitro* differentiation conditions, the proportion of IL-17A-producing memory CD4^+^ T cells and levels of IL-17A production by these cells were similar between the patient and healthy controls (Fig. 6C, 6D). Nevertheless, production of IFN-γ and TNF-a by memory CD4^+^ T cells was lower in the patient than in healthy donors (Fig. 6A, S6A). Finally, although proportions of Th2 phenotype cells in the patient were similar to those in healthy controls, production of Th2 cytokines secreted by memory T cells were higher (Fig. 6B, S6B). Overall, distribution of leukocyte subsets and CD4^+^ T-cell differentiation and function *in vivo* and *ex vivo* in our patient were largely within the dynamic ranges observed for CD4^+^ T cells from healthy donors, suggesting only a very modest, if any, effect of IL-17RC deficiency on the behavior and function of these cells.

## Discussion

We report the fourth patient and the first Asian patient with AR IL-17RC deficiency due to a homozygous novel duplication variant of *IL17RC*. Consistent with previously reported IL-17RC-deficient patients (30), this patient had early-onset mucocutaneous candidiasis but without any other severe infectious diseases, including mucocutaneous *S. aureus* disease or autoimmune clinical signs. This novel variant causes duplication of 46 or 49 bp within exon 13, leading to a frameshift and a premature stop codon at position 457. The patient’s fibroblasts exhibited a complete lack of *CXCL1* or *IL6* upregulation after IL-17A stimulation, though this defect was restored by introduction of the WT *IL17RC* allele. We therefore conclude that the homozygous *IL17RC* variant identified in this patient with isolated CMC is a novel pathogenic variant leading to AR IL-17RC deficiency. These results support that IL-17A/F signaling mediated by IL-17RC is essential for mucocutaneous defense against *Candida* but is otherwise redundant for immunity against other pathogens.

Patients with AR IL-17RA or AR ACT1 deficiency frequently develop staphylococcal skin and mucosal infections. Hence, IL-17 immunity is essential for mucocutaneous defense not only against *Candida* but also against *S. aureus* (27–29). On the other hand, mucocutaneous staphylococcal infections were not detected in three previously identified patients (30) or the present patient with AR IL-17RC deficiency. The mechanism for this phenotypic difference remains unclear. Abnormal IL-17E immunity, which is affected in AR IL-17RA deficiency but preserved in AR IL-17RC deficiency, has been suggested but not demonstrated as a plausible mechanism to explain this phenotypic difference (30). Three patients from three generations with CMC, mucocutaneous *S. aureus* infection, and a connective tissue disorder due to AD deficiency of c-Jun N-terminal kinase 1 (JNK1), a component of the MAPK signaling pathway, were recently reported (18). These patients displayed impaired responses to IL-17A and IL-17F and low proportions of Th17 cells but intact responses to IL-17E. A recent study suggested that heterodimers of IL-17RA and IL-17RD function as alternative receptor subunits of IL-17A in primary mouse and human keratinocytes (48). IL-17A signaling is mediated by both IL-17RC and IL-17RD, and the IL-17RA/IL-17RD pathway mainly activates p38 MAPK and JNK (48). These findings suggest that the IL-17RC-independent IL-17RA/IL-17RD pathway may provide the IL-17 immune activity required for protection against infection by *S. aureus* in IL-17RC-deficient patients. Regardless, additional research is needed to fully understand the precise role of IL-17 immunity in mucocutaneous defense against *S. aureus*.

To date, no system has been developed for qualitative evaluation of *IL17RC* variants *in vitro*. In this study, we successfully established a precise *in vitro* assay system using a CRISPR-generated *IL17RC*-knockout HeLa cell line. The system was able to accurately and unambiguously distinguish loss-of-function variants from neutral variants found in public databases and predicted to be functionally intact. In general, increased identification of rare variants through comprehensive approaches in genomic research has emphasized the importance of artificial evaluation systems that accurately assess the impact of variants in the absence of patient-derived samples. In particular, evaluation of *IL17RC* variants requires patient-derived fibroblasts, making an artificial system even more important. Diagnosis based on accurate evaluation of pathogenicity for identified variants allows for appropriate management and/or treatment of the patient.

AR IL-17RA deficiency is the major genetic cause in patients with isolated CMC due to impaired IL-17 immunity, with 28 cases reported to date. However, only four cases of AR IL-17RC deficiency, including the current case, have been reported thus far. Evolutional and epidemiological genetics analyses revealed no obvious difference in the strength of negative selection at the *IL17RA* and *IL17RC* loci. However, in the general population (gnomAD), the cumulative frequency of pLOF variants is higher for *IL17RC* than *IL17RA*, inconsistent with the fact that the number of patients with AR IL-17RA deficiency identified thus far is higher than that with AR IL-17RC deficiency. These results thus do not explain the observed difference in the incidence of IL-17RA and IL-17RC deficiency. Given that a homozygous p.Q378* variant of *IL17RC* is included in a public database, the presence of undiagnosed mild cases or cases with incomplete penetrance may be expected for AR IL-17RC deficiency. In contrast, no homozygous deleterious variant of *IL17RA* was found in the public database, suggesting higher penetrance for AR IL-17RA deficiency. If this hypothesis is true, some cases of AR IL-17RC deficiency may not be diagnosed. Further study is needed to fully characterize the cellular, clinical, and frequency differences between AR IL-17RA and IL-17RC deficiencies.

## Figures and Tables

**Figure 1:** A CMCD patient with AR IL-17RC deficiency. **A,** Pedigree of the family established by *IL17RC*genotyping. A sporadic case of CMCD without a family history of CMCD. The proband is indicated by an arrow. The black symbol represents an individual with CMCD. “E?” indicates individuals for whom genetic analysis was not possible. **B**, Pictures of the oral and esophageal candidiasis of the patient. The left picture represents oral candidiasis at the age of seven years, and the right picture represents esophageal candidiasis at the same age. **C**, Panel testing of next-generation sequencing (NGS). Schematic representation indicating an increased number of reads in a region including the entire exon 13 of *IL17RC* by IGV software. **D**, Sanger sequencing chromatograms of the *IL17RC* region harboring a duplication variant (Chr3: 9,971,476–9,971,606 dup (+131bp)) for the proband and family members. **E,** Protein-level representations of the patient’s variant for both duplications of 46 and 49 bases (p.D458Afs*16 and p.D458Afs*17). **F,**Schematic representation of the IL-17RC protein, with its signal sequence (SS), extracellular (EC), transmembrane (TM), intracellular (IC), and SEFIR (similar expression to fibroblast growth factor genes and IL-17R) domains and the positions affected by variants (our variant is shown in red). **G,** Predicted CADD scores (GRCh37-v1.6, https://cadd.gs.washington.edu/) and global allele frequencies of *IL17RC*variants, for which homozygotes are reported in the gnomAD database (v2.1.1). A red dot shows our patient’s variant (CADD score 33); blue dots show previously reported *IL17RC*-LOF variants.

## Data Availability

The datasets generated during and/or analysed during the current study are available from the corresponding author on reasonable request.

## Abbreviations

CMC: Chronic mucocutaneous candidiasis
AD: Autosomal dominant
AR: Autosomal recessive
IL: Interleukin
Th: T helper
Tfh: T follicular helper
IEI: Inborn errors of immunity
NGS: Next generation sequencing
WT: Wild-type
SEFIR: Similar expression to fibroblast growth factor genes and IL-17R
pLOF: Predicted to be loss-of-function
